# Artificial Intelligence in Hypertension Management: Promise Must Precede Practice

**DOI:** 10.7759/cureus.111109

**Published:** 2026-06-18

**Authors:** Kunj R Ghantiwala, Ruchik Kevadiya, Pradeep Pentapurthy, Arpitkumar R Thakor, Sarwan Kumar

**Affiliations:** 1 Medicine, Gujarat Medical Education and Research Society (G.M.E.R.S.) Medical College, Navsari, Navsari, IND; 2 Medicine, Government Medical College, Bhavnagar, Bhavnagar, IND; 3 Internal Medicine, Henry Ford Health System, Rochester Hills, USA; 4 Internal Medicine, Wayne State University/Henry Ford Rochester Hospital, Rochester Hills, USA; 5 Medicine, Saint Martinus University Faculty of Medicine, Willemstad, CUW; 6 Medicine, Larsen and Toubro Health and Dialysis Centre, Surat, IND; 7 Internal Medicine, Wayne State University School of Medicine, Rochester Hills, USA

**Keywords:** artificial intelligence, blood pressure monitoring, cardiovascular disease, hypertension, machine learning

## Abstract

Hypertension remains one of the most important modifiable risk factors for cardiovascular disease worldwide, yet blood pressure control rates remain suboptimal despite advances in diagnosis and treatment. Artificial intelligence (AI) has emerged as a promising tool to improve hypertension care through enhanced risk prediction, continuous monitoring, and clinical decision support. Enthusiasm surrounding AI must be balanced against challenges related to algorithmic bias, model interpretability, data quality, and clinical implementation. While AI has the potential to transform hypertension management, its greatest challenge is no longer predictive accuracy but successful integration into real-world clinical practice. This editorial discusses the opportunities and limitations of AI in hypertension care and argues that implementation science, transparency, and equitable deployment should become the next priorities for the field.

## Editorial

Hypertension affects more than one billion people globally and remains a leading cause of cardiovascular morbidity and mortality. Despite the availability of effective pharmacologic therapies and evidence-based clinical guidelines, a substantial proportion of patients remain undiagnosed, untreated, or inadequately controlled. These persistent gaps have prompted growing interest in innovative technologies that enable earlier detection, more accurate risk stratification, and individualized treatment approaches [1].

Artificial intelligence (AI) has sparked a lot of interest in these breakthroughs. AI systems can examine huge, complicated datasets, find hidden links, and produce predictions that may outperform conventional statistical methods by utilizing machine learning and deep learning techniques. AI has quickly developed over the past few years from a research idea to a potentially revolutionary tool for cardiovascular health, especially in the treatment of hypertension. [1].

The strongest evidence supporting AI in hypertension care comes from risk prediction. Traditional prediction models often rely on a limited number of variables and assume relatively simple relationships among risk factors. In contrast, the machine learning algorithms can incorporate a broad range of demographic, clinical, laboratory, and behavioral data while identifying complex interactions among variables. A systematic review and meta-analysis by Chowdhury et al. found that machine learning models generally demonstrated superior predictive performance compared with conventional regression-based approaches for hypertension prediction [2]. Similarly, Hwang et al. validated machine learning algorithms in large nationwide cohorts and reported strong performance in predicting incident hypertension using routinely collected health screening data [3].

These findings suggest that AI may enable a shift from reactive management to proactive prevention. Earlier identification of persons at a high risk of developing hypertension in the future could facilitate targeted lifestyle interventions, closer monitoring, and timely therapeutic strategies before cardiovascular complications develop. From a population health perspective, this may represent one of the most valuable contributions of AI to hypertension care.

However, predictive accuracy alone does not guarantee clinical impact. A recurring challenge in healthcare AI is the gap between model performance in research settings and effectiveness in the real world while treating patients. Many published algorithms are developed using retrospective datasets under highly controlled conditions. Whether these models maintain comparable performance across diverse healthcare systems, ethnic groups, and socioeconomic settings remains uncertain. Consequently, the field may be approaching a point where implementation rather than innovation becomes the primary challenge.

AI also offers opportunities to improve blood pressure monitoring and disease detection. Traditional office-based blood pressure measurements provide only intermittent assessments and are often affected by white-coat hypertension, masked hypertension, and measurement variability. AI-enhanced remote monitoring systems and wearable technologies may provide a more comprehensive understanding of blood pressure patterns over time [1]. Such approaches have the potential to improve patient engagement while supporting more personalized treatment plans.

Perhaps even more intriguing are emerging applications that move beyond conventional blood pressure measurement. Sau et al. demonstrated that deep learning analysis of electrocardiographic data could identify individuals at an increased risk of developing hypertension in the future before clinical diagnosis [4]. This capability raises the possibility that AI may detect subtle physiologic changes that precede overt disease, creating opportunities for earlier intervention and prevention.

The integration of AI into clinical decision-making represents another important area of development. Using electronic health record data, Nguyen et al. developed predictive models capable of identifying patients at risk of sustained uncontrolled hypertension and hypertensive crises [5]. Such tools may help clinicians prioritize high-risk patients, allocate resources more efficiently, and optimize treatment strategies. Nevertheless, the value of these systems ultimately depends on their ability to integrate more effectively into existing clinical workflows in patient management without increasing complexity.

One of the chronic conditions that might benefit most from the application of AI is hypertension. Through routine blood pressure checks, laboratory studies, medication records, wearable technology, and electronic health records, hypertension produces vast amounts of structured and easily accessible data, in contrast to many complex cardiovascular conditions that call for sophisticated imaging or invasive testing. Because hypertension is chronic, it can be continuously monitored and evaluated over time, which makes it a perfect setting for machine learning applications. Furthermore, even small gains in the identification and management of hypertension may result in significant population-wide decreases in cardiovascular events. Because of these factors, hypertension is a feasible and potentially significant target for the practical use of AI-driven healthcare solutions.

The future of AI in hypertension management will depend less on predictive accuracy and more on clinical trust. Algorithms that function as “black boxes” may achieve impressive performance but are unlikely to gain widespread acceptance among healthcare professionals. The next generation of AI tools should prioritize transparency, interpretability, and meaningful clinical impact rather than incremental gains in statistical performance.

Despite its promise, several barriers continue to limit the widespread adoption of AI in routine clinical practice. Algorithmic bias remains a concern because models developed from nonrepresentative datasets may perform inconsistently across diverse populations. Additional challenges include data privacy, regulatory oversight, integration into existing clinical workflows, and the need for prospective validation studies demonstrating real-world effectiveness [1]. Importantly, AI should be viewed as an adjunct to, not a replacement for, clinical judgment. Effective hypertension management requires consideration of patient preferences, comorbidities, and social determinants of health that cannot be fully captured by algorithms alone.

Conclusion

AI has demonstrated a great deal of promise for managing hypertension, especially in the areas of clinical decision support, disease detection, and risk prediction. According to available data, AI can detect high-risk individuals, enhance monitoring techniques, and enable more individualized care approaches. However, resolving issues with transparency, equity, validation, and implementation will be more crucial to AI's future success in managing hypertension than attaining slight gains in predictive performance. AI has the potential to play a crucial role in the treatment of hypertension in the future with proper supervision and ongoing innovation.
